# TSPAN5 influences serotonin and kynurenine: pharmacogenomic mechanisms related to alcohol use disorder and acamprosate treatment response

**DOI:** 10.1038/s41380-020-0855-9

**Published:** 2020-08-04

**Authors:** Ming-Fen Ho, Cheng Zhang, Lingxin Zhang, Lixuan Wei, Ying Zhou, Irene Moon, Jennifer R. Geske, Doo-Sup Choi, Joanna Biernacka, Mark Frye, Zhexing Wen, Victor M. Karpyak, Hu Li, Richard Weinshilboum

**Affiliations:** 1 Department of Molecular Pharmacology and Experimental Therapeutics, Mayo Clinic, 200 First Street SW, Rochester, MN 55905 USA; 2 Department of Cell Biology, Emory University, 615 Michael Street, Atlanta, GA 30322 USA; 3 Department of Health Sciences Research, Mayo Clinic, 200 First Street SW, Rochester, MN 55905 USA; 4 Department of Psychiatry and Psychology, Mayo Clinic, 200 First Street SW, Rochester, MN 55905 USA

**Keywords:** Molecular biology, Cell biology

## Abstract

We previously reported that SNPs near *TSPAN5* were associated with plasma serotonin (5-HT) concentrations which were themselves associated with selective serotonin reuptake inhibitor treatment outcomes in patients with major depressive disorder (MDD). *TSPAN5* SNPs were also associated with alcohol consumption and alcohol use disorder (AUD) risk. The present study was designed to explore the biological function of *TSPAN5* with a focus on 5-HT and kynurenine concentrations in the tryptophan pathway. Ethanol treatment resulted in decreased 5-HT concentrations in human induced pluripotent stem cell (iPSC)-derived neuron culture media, and the downregulation of gene expression of *TSPAN5*, *DDC, MAOA, MAOB, TPH1*, and *TPH2* in those cells. Strikingly, similar observations were made when the cells were treated with acamprosate—an FDA approved drug for AUD therapy. These results were replicated in iPSC-derived astrocytes. Furthermore, TSPAN5 interacted physically with proteins related to clathrin and other vesicle-related proteins, raising the possibility that *TSPAN5* might play a role in vesicular function in addition to regulating expression of genes associated with 5-HT biosynthesis and metabolism. Downregulation of *TSPAN5* expression by ethanol or acamprosate treatment was also associated with decreased concentrations of kynurenine, a major metabolite of tryptophan that plays a role in neuroinflammation. Knockdown of TSPAN5 also influenced the expression of genes associated with interferon signaling pathways. Finally, we determined that *TSPAN5* SNPs were associated with acamprosate treatment outcomes in AUD patients. In conclusion, *TSPAN5* can modulate the concentrations of 5-HT and kynurenine. Our data also highlight a potentially novel pharmacogenomic mechanism related to response to acamprosate.

## Introduction

We previously reported that *TSPAN5* eQTL SNPs on chromosome 4 were associated with variation in plasma serotonin (5-HT) concentrations which were themselves correlated with selective serotonin reuptake inhibitor treatment outcomes in patients with major depressive disorder (MDD) [1]. We also reported that knockdown of TSPAN5 resulted in the downregulation of genes involved in both 5-HT biosynthesis and metabolism [1]. It should be pointed out that depression is the most common psychiatric co-morbidity among AUD patients [2–4]. A recent genome-wide association study (GWAS) of alcohol consumption in UK Biobank participants identified a series of genome-wide significant variants on chromosome 4 [5]. Strikingly, several of those SNPs (rs3114045, rs193099203 and rs9991733) are *trans*-eQTLs in the brain for *TSPAN5* which maps to chromosome 4. When the UK Biobank study results were stratified by sex, the rs114026228 SNP in *TSPAN5* (*p* = 3.60E−13) was the top signal associated with alcohol consumption in men [5]. In addition, a recent GWAS meta-analysis demonstrated that *TSPAN5* SNPs were associated with alcohol use disorder (AUD) risk in an African–American population [2]. It should also be pointed out that the *TSPAN5* rs11947402 SNP which was originnally identified from our GWAS for baseline plasma 5-HT concentrations in MDD patients was also associated with AUD risk (*p* = 0.017) in that same AUD GWAS meta-analysis [2, 6]. As a result of this growing body of evidence that TSPAN5 may play a role in both MDD and AUD risk, the present study was designed to explore the biological function of *TSPAN5* with a focus on the tryptophan pathway using human iPSC-derived CNS cells exposed to either ethanol (EtOH) or acamprosate—an FDA approved medication for the treatment of AUD [7]. The significance of the present study results from this expanding body of molecular genomic data with regard to TSPAN5, from the societal importance of AUD, from the possibility of more highly individualized treatment for AUD, and from the fact that the results of the studies described subsequently suggest novel genetic mechanisms that might influence individual variation in acamprosate response in AUD patients [8–10].

**Fig. 1:**
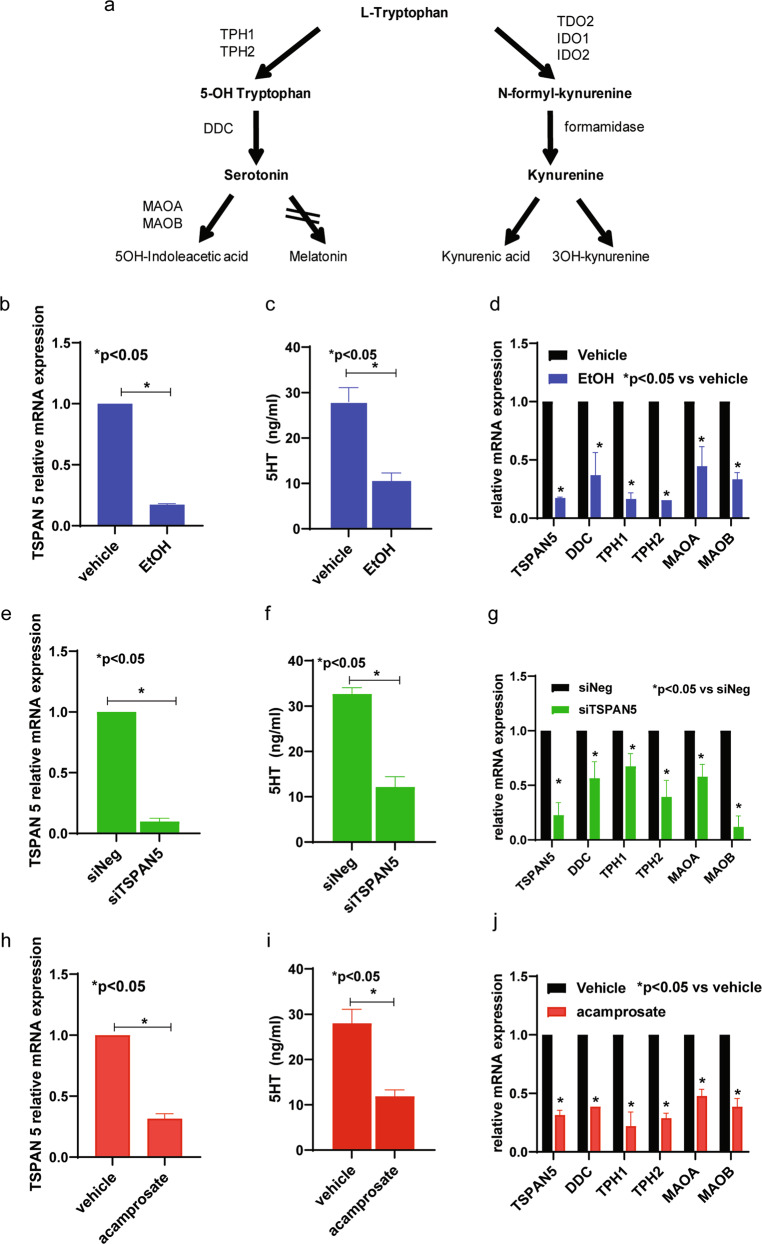
TSPAN5 and 5-HT concentrations can be regulated by EtOH and acamprosate. **a** Tryptophan metabolic pathway in schematic outline. **b** TSPAN5 mRNA expression was down-regulated in iPSC-derived forebrain neurons (*n* = 5) after exposure to EtOH (25 mM) for 24 h. **c** 5-HT concentrations decreased significantly in iPSC-derived forebrain neuron culture medium in response to EtOH treatment. **d** Downregulation of TSPAN5 expression resulted from EtOH exposure, with downstream effects on the expression of a series of genes involved in 5-HT biosynthesis and metabolism. In parallel, knockdown of TSPAN5 (**e**) decreased 5-HT concentrations (**f**), and resulted in the downregulation of genes associated with 5-HT biosynthesis and metabolism (**g**). Similar results were obtained when the cells were exposed to acamprosate (5 μM) for 24 h. Specifically, TSPAN5 expression, 5-HT concentration and expression of the genes displayed in panels (**c**, **d**, **f**, and **g)** all were down-regulated in response to acamprosate treatment (**h**–**j**). Five independent human-derived-iPSC-derived neuron cell lines were used to perform these experiments. Cells were seeded in 6-well plates at a density of 2.5 ×10^5^ cells per well in 2 ml culture medium. *A *p* value ≤ 0.05 was considered statistically significant (two tailed paired *t* test). Three independent experiments were performed. All values are mean ± SEM.

*TSPAN5* is one of the 33 members of the tetraspanin gene family [11]. *TSPAN5* is widely expressed in the brain based on the GTEx database [12]. However, the possible functional role of *TSPAN5* in AUD is unknown. Since our previous report demonstrated that *TSPAN5* is associated with plasma concentrations of 5-HT—a metabolite of tryptophan—the present study places a focus on the impact of *TSPAN5* on the tryptophan metabolic pathway, one branch of which leads to the formation of 5-HT, with the other main branch resulting in the formation of kynurenine (Fig. 1a). During our previous MDD study we found that kynurenine was, among the metabolites assayed, the most highly associated with severity of depression symptoms [1]. In an attempt to understand the possible roles of *TSPAN5*, EtOH and acamprosate in the regulation of 5-HT biosynthesis and metabolism, we first demonstrated that both EtOH and acamprosate decreased 5-HT in the culture medium of iPSC-derived forebrain neurons and astrocytes. In parallel we demonstrated that TSPAN5 expression was also down-regulated in the presence of EtOH or acamprosate. We also demonstrated that *TSPAN5* could regulate both kynurenine concentrations and the expression of a series of genes associated with interferon (IFN) related pathways. Those experiments were followed by a series of functional genomic studies using astrocytes and microglia which showed that *TSPAN5* might have a role in CNS immune response. Finally, we found that several SNPs that are *cis*-eQTLs for *TSPAN5* were associated with acamprosate treatment outcomes in patients with AUD.

In summary, the present study greatly extends our original observations with regard to *TSPAN5* and plasma 5-HT regulation [1], and serves to highlight a novel pharmacogenomic mechanism related to acamprosate treatment response by which *TSPAN5* can modulate and influence major metabolites of tryptophan and the CNS immune response—both of which have been implicated in neuropsychiatric disorders, including AUD [13]. Taken together, these observations serve to emphasize the possible importance of *TSPAN5* in acamprosate treatment response. As a result, they have expanded and broadened our understanding of acamprosate’s mechanism of action.

## Methods and materials

### Subjects

The Mayo Clinic Center for the Individualized Treatment of Alcoholism recruited 442 AUD subjects with associated clinical data, and DNA samples were obtained for genotyping [14–16]. Specifically, 305 European-American subjects included in this study had acamprosate treatment outcomes available, i.e. abstinence length during acamprosate therapy [17]. In addition, induced pluripotent stem cells (iPSCs) were generated from five healthy subjects from the Mayo Clinic Biobank. All subjects provided written informed consent for their participation in these studies. The protocol for this study was reviewed and approved by the Mayo Clinic Institutional Review Board (reference number: 10-006845). See Supplementary text for details.

### Generation of patient-derived iPSCs, and glial and neuronal cells differentiation

Fibroblasts from skin biopsies for all subjects were utilized for iPSC reprogramming using the CytoTune™-iPS 2.0 Sendai Reprogramming Kit (A16517, Thermo Fisher, USA). Patient-derived iPSCs were characterized as previously described [18, 19]. Those iPSCs were then differentiated into astrocytes and forebrain neurons as previously described [20]. Cells were then treated with various concentrations of EtOH or acamprosate within the range of concentrations observed in patients drinking EtOH or patients treated with acamprosate observed during acamprosate treatment of patients with AUD, respectively [21]. See Supplementary text for details.

### RNA Sequencing and functional genomic studies

RNA-seq was performed by GENEWIZ using an Illumina HiSeq 4000 platform. Fastq files containing paired RNASeq reads were aligned with STAR [22] against the UCSC human reference genome (hg19) using Bowtie 2.2.3 with default settings [23]. Gene level counts from uniquely mapped, non-discordant read pairs were obtained using the subRead featureCounts program (v1.4.6) [24] and gene models from the UCSC hg19 Illumina iGenomes annotation package. Differential expression analysis was performed using the DESeq2 package with default parameters [25]. Gene set enrichment analysis (GSEA) software was used for pathway analysis [26, 27]. Real time PCR was used for validation and primer sets for real time PCR are listed in Supplementary Table 1. We performed functional genomic studies including high-performance liquid chromatography, immunofluorescence staining and confocal imaging analysis, mass spectrometry, Western blot analysis, TSPAN5 siRNA knockdown and CRISPR/cas9 knockout studies. See the Supplementary text for details.

## Results

### ***TSPAN5*is an alcohol responsive gene**

As a first step, we set out to determine the possible effect of EtOH on TSPAN5 expression and concentrations of 5-HT—one of the metabolites of tryptophan (Fig. 1a) —using iPSC-derived forebrain neurons. TSPAN5 expression was significantly down-regulated in iPSC-derived forebrain neurons after EtOH (25 mM) exposure that is considered physiologically relevant, with 25 mM EtOH being slightly higher than the 0.08% blood alcohol concentration (BAC) required to be legally intoxicated in most states in the United State [28] (Fig. 1b). In parallel, 5-HT concentrations in the culture media decreased substantially after EtOH exposure (Fig. 1c). We also observed that the downregulation of TSPAN5 by EtOH was associated with decreased mRNA expression of *DDC*, *TPH1*, *TPH2, MAOA*, and *MAOB*, all of which play roles in 5-HT biosynthesis and metabolism (Fig. 1d). In followup of this observation, siRNA knockdown studies were performed using four independent TSPAN5 siRNAs as well as one pooled siRNA (Dharmacon Chicago, IL, USA). The results for all of those experiments were consistent. Specifically, knockdown of TSPAN5 in iPSC-derived neurons to 5% of its baseline significantly decreased 5-HT concentrations in the culture media (Fig. 1e, f), with associated downregulation of the expression of *DDC*, *TPH1*, *TPH2, MAOA*, *MAOB* (Fig. 1g), consistent with our previous report that used a neuroblastoma cell line, SK-N-BE(2) [1]. Use of that neuroblastoma cell line also made it possible for us to optimize treatment conditions for our subsequent functional genomic studies, which would have been impractical using iPSC-derived neurons due to their high cost and the length of time required for their differentiation (see Supplementary Fig. 1).

### **Acamprosate modulates** TSPAN5 **expression and 5-HT concentrations**

The next series of experiments was performed to determine whether acamprosate, an FDA approved drug for the treatment of AUD, might also influence TSPAN5 expression and 5-HT concentrations in iPSC-derived neuron culture medium. The concentrations of acamprosate used to perform those experiments were selected to fall within the range of blood drug concentrations observed during acamprosate therapy of patients with AUD [21]. Strikingly, TSPAN5 expression in iPSC-derived forebrain neurons was also down-regulated in the presence of acamprosate (Fig. 1h). In parallel, 5-HT concentrations in the neuron culture medium also decreased significantly (Fig. 1i). Even more striking, and somewhat surprisingly, we observed that expression of the genes associated with monoamine neurotransmitter biosynthesis and metabolism shown in Fig. 1d, g was also down-regulated in response to acamprosate treatment (Fig. 1j).

### ***TSPAN5*biological function in** iPSC**-derived astrocytes**

**Fig. 2:**
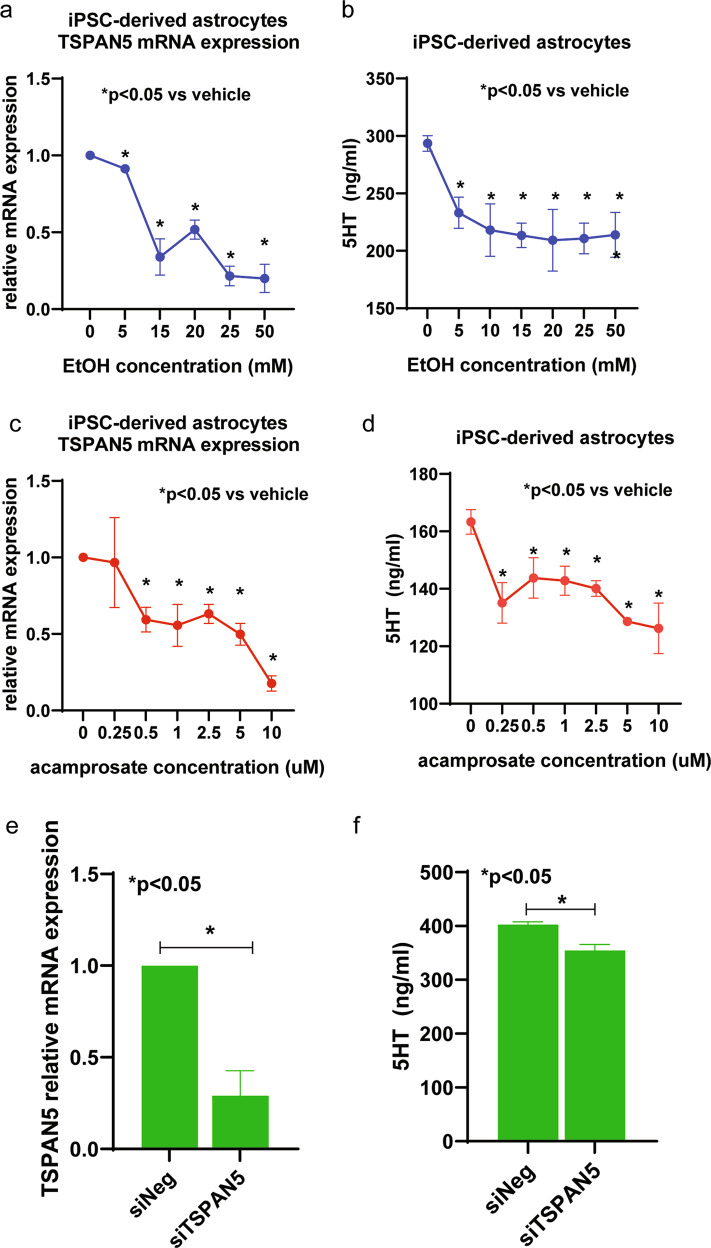
Effects of EtOH and acamprosate on iPSC-derived astrocytes. **a** Downregulation of TSPAN5 mRNA expression (*F*_4,30_ = 24.25, *p* = 0.0007) and (**b**) 5-HT concentrations (*F*_4,35_ = 4.46, *p* = 0.046) in iPSC-derived astrocyte culture medium (*n* = 5) in the presence of EtOH (from 5 mM to 50 mM) for 24 h. **c** Downregulation of TSPAN5 mRNA expression (*F*_4,35_ = 4.54, *p* = 0.037) and (**d**) 5-HT concentrations (*F*_4,35_ = 4.65, *p* = 0.031) in iPSC-derived astrocyte culture medium (*n* = 5) in the presence of acamprosate (0.25–10 µM) for 24 h. **e** Knockdown of TSPAN5 (**f**) decreased the 5-HT concentrations in iPSC-derived astrocyte culture medium. Five independent human-derived-iPSC-derived astrocyte cell lines were used to perform these experiments. Cells were seeded in T75 culture flasks at a density of 2.5 × 10^6^ cells. *A *p* value ≤ 0.05 was considered statistically significant. Three independent experiments were performed. All values are mean ± SEM.

Having determined the effect of EtOH and acamprosate on forebrain neurons, we next determined the effect of EtOH and acamprosate on iPSC-derived astrocytes. TSPAN5 expression also decreased significantly in response to EtOH treatment of iPSC-derived astrocytes (Fig. 2a). Consistently, 5-HT concentrations were also significantly decreased in iPSC-derived astrocyte culture medium in the presence of EtOH (Fig. 2b). Similar results were observed when cells were treated with acamprosate (Fig. 2c, d). In addition, knockdown of TSPAN5 in iPSC-derived astrocytes significantly decreased 5-HT concentrations in the culture medium (Fig. 2e, f). We should point out that iPSC-derived astrocyte cultures displayed about 10-fold higher concentrations of 5HT than did the neurons, as shown in Fig. 1. That is because the cell numbers used to perform assays for the iPSC-derived astrocytes were at least 10 times higher than for the iPSC-derived neurons, as described in the figure legend. It was not practically possible to use higher numbers of iPSC-derived neurons as a result of their high cost and the length of time required for their differentiation. It should also be emphasized that, unlike iPSC-derived neurons, iPSC-derived astrocytes can be expanded for functional studies that require a larger number of cells. Using the required larger number of cells, we next performed TSPAN5 pulldown studies with iPSC-derived astrocytes for mass spectrometric identification of the proteins “pulled down” and identified a series of proteins that included clathrin heavy chain and other neurotransmitter vesicle-related proteins including AP2M1, AP3M1, VAMP7 and VPS29, all of which interacted physically with TSPAN5, (Supplementary Table 2). These observations suggested that *TSPAN5* might play roles in both the regulation of 5-HT biosynthesis and metabolism (see Fig. 1 and Fig. 2) as well as in vesicular function.

### TSPAN5 influences kynurenine

**Fig. 3:**
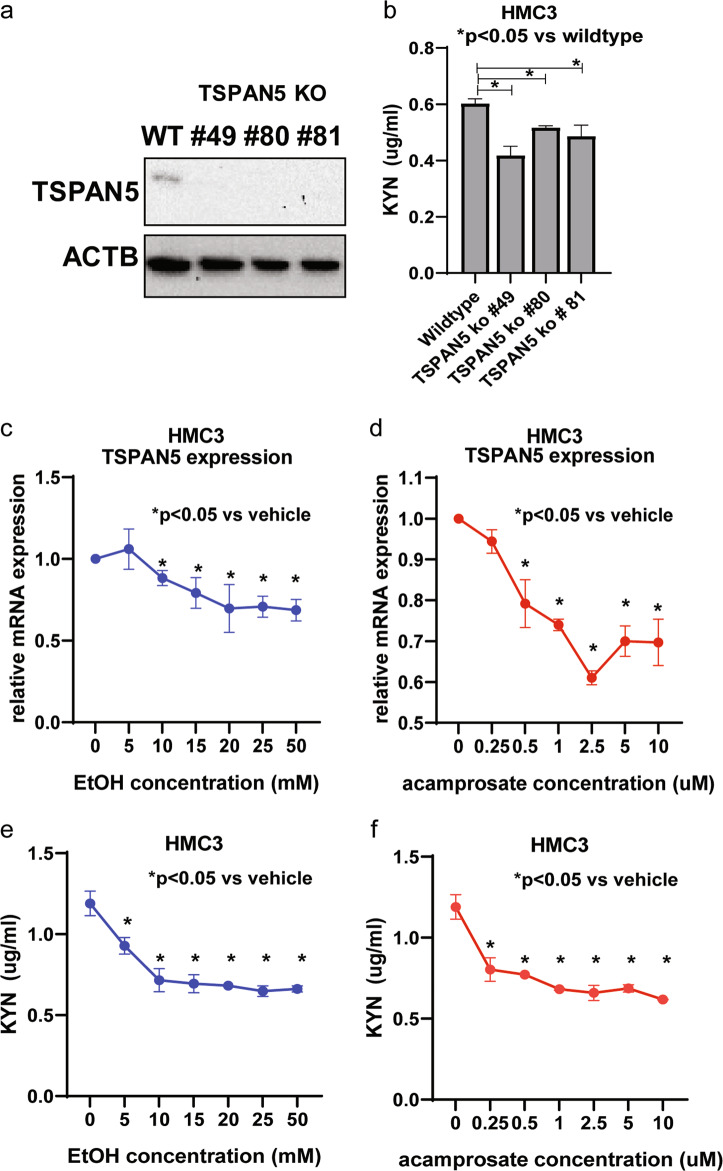
TSPAN5 regulates kynurenine concentrations. **a** Western blot analysis of *TSPAN5* knockout clones for HMC3 cells. **b** Lower kynurenine concentrations (*F*_3,12_ = 10.54, *p* = 0.013) were observed in TSPAN5 knockout cells. **c**–**d** TSPAN5 mRNA expression was down-regulated in response to EtOH (*F*_2,21_ = 5.8, *p* = 0.018) or acamprosate treatment (*F*_2,21_ = 14.87, *p* = 0.0011) of HMC3 cells. **e** In parallel, kynurenine concentrations displayed significant decreases in the presence of EtOH (*F*_2,21_ = 15.8, *p* = 0.0009) or acamprosate (*F*_2,21_ = 19.24, *p* = 0.0005) for 24 h. *p ≤ 0.05, as compared to wildtype cells or no drug treatment. Three independent experiments were performed. All values are mean ± SEM.

We next set out to determine whether TSPAN5 might influence kynurenine concentrations (Fig. 1a) just as it did for another major tryptophan pathway metabolite, 5-HT, and whether TSPAN5 downregulation resulting from EtOH or acamprosate treatment might also affect kynurenine concentrations. To answer those questions, we used HMC3 cells, a human microglial cell line, to perform the functional studies because microglia are the major cell type in the CNS responsible for immune response [29] and because our previously published studies had placed a focus on the relationship of the “kynurenine arm” of tryptophan metabolism with immunity and inflammation [1]. Specifically, we knocked out TSPAN5 using CRISPR-Cas9 and demonstrated that kynurenine concentrations decreased significantly in the TSPAN5 knockout cell culture medium as compared to wildtype cells (Fig. 3a, b). In line with those observations, both TSPAN5 expression and kynurenine concentrations (Fig. 3c, f) were decreased in response to EtOH or acamprosate treatment. These results significantly extended our original observations with regard to TSPAN5 and plasma 5-HT regulation [1], and served to highlight a possible novel pharmacogenomic mechanism by which TSPAN5 can influence kynurenine concentrations which have been implicated in CNS immune response and neuropsychiatric disorders.

### TSPAN5 may also play a role in CNS immune response

**Fig. 4:**
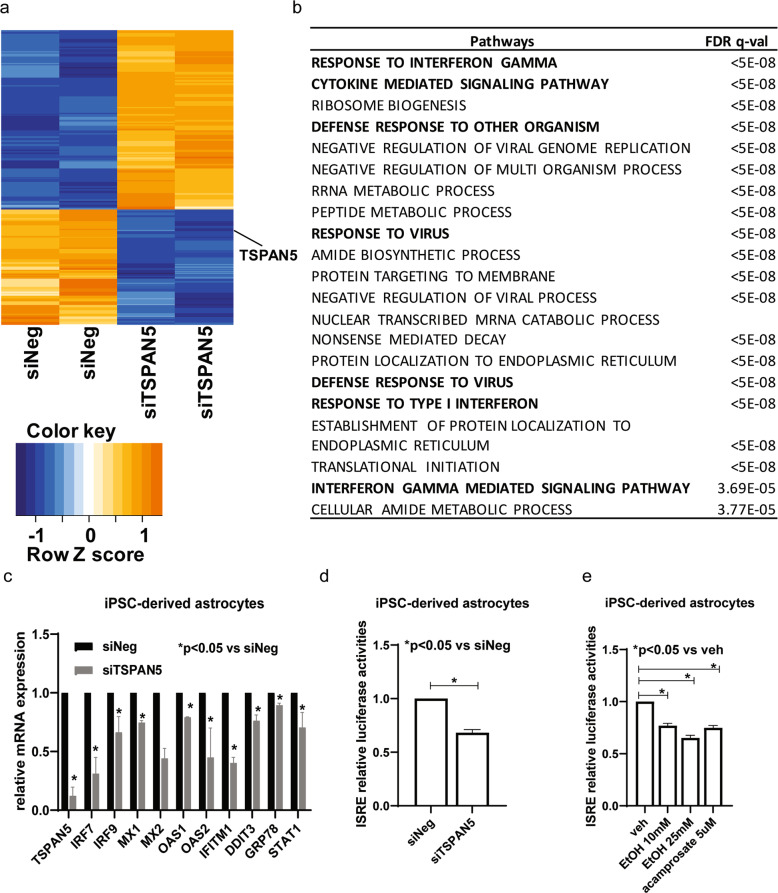
*TSPAN5* expression plays a role in immune response. **a** Heat map showing expression profiles for 301 genes (FDR ≤ 0.05) with mRNA expression that was altered after TSPAN5 knockdown as determined by RNA-seq. Two biological replicates were performed. **b** Pathway analysis was performed using gene set enrichment analysis (GSEA) software, and those data placed a focus on the interferon pathways and the immune response—highlighted in the figure. **c** mRNA expression of genes involved in the IFN signaling pathways in iPSC-derived astrocytes before or after TSPAN5 knockdown, as determined by real time PCR with five independent human iPSC-derived astrocytes. **d** Knockdown of TSPAN5 decreased ISRE luciferase activities. **e** ISRE luciferase activities could be regulated by EtOH or acamprosate (*F*_4,15_ = 52.05, *p* = 0.0012). *p ≤ 0.05, as compared to negative siRNA or vehicle treatment. Three independent experiments were performed. All values are mean ± SEM.

We also used iPSC-derived astrocytes to perform mRNA expression profiling before and after TSPAN5 knockdown, and identified 301 genes that displayed significant changes in expression (FDR ≤ 0.05) after the knockdown of TSPAN5 (Fig. 4a, and Supplementary Table 3). Pathway enrichment analysis demonstrated that a series of immune response signaling pathways were the most common and most highly affected pathways after TSPAN5 knockdown (Fig. 4b, and Supplementary Table 4). Those findings were validated using four additional human iPSC-derived astrocytes. Specifically, we confirmed that genes associated with the “response to interferon (IFN)” pathway were down-regulated when TSPAN5 was knocked down (Fig. 4c). We then tested the effect of TSPAN5 on IFN signaling pathways using an IFN-stimulated response element (ISRE) luciferase reporter system. Knockdown of TSPAN5 in iPSC-derived astrocytes resulted in significantly decreased ISRE activity (Fig. 4d). That was also true when iPSC-derived astrocytes were treated with either EtOH or acamprosate (Fig. 4e). We then replicated those findings using HMC3 cells. Consistently, ISRE luciferase activities were significantly lower in TSPAN5 knockout cells than in wildtype cells (Supplementary Fig. 3a). TSPAN5 in HMC3 cells was also involved in differences in expression for a panel of genes involved in IFN signaling pathways as shown in Fig. 4c. Specifically, TSPAN5 knockout cells displayed significantly decreased levels of expression for IRF7, IRF9, MX1, MX2, OAS1, OAS2, IFITM1, DDIT3, GRP78, and STAT1 (Supplementary Fig. 3b). In similar fashion, treatment of HMC3 cells with either EtOH or acamprosate resulted in significantly decreased expression of genes associated with response in IFN signaling pathways (Supplementary Fig. 3c, d). These findings suggested that both acamprosate and EtOH could influence *TSPAN5* gene expression which, in turn, altered both 5-HT and kynurenine concentrations and down-regulated IFN signaling pathways in the brain—all of which might have implications for neuropsychiatric disorders such as AUD. Therefore, the next series of studies was designed to determine the possible association of *TSPAN5* genetic variants with acamprosate treatment response in AUD patients.

### ***TSPAN5*SNPs are associated with** response **to acamprosate treatment**

**Fig. 5:**
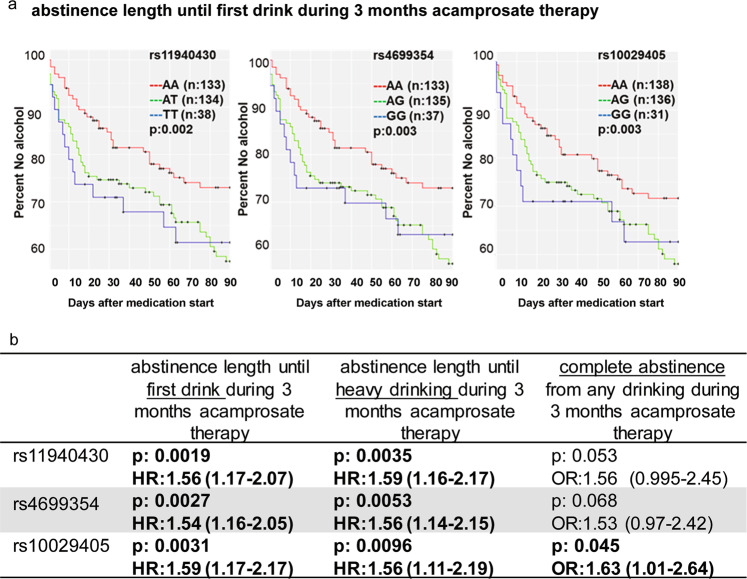
*TSPAN5* SNPs were associated with acamprosate treatment response. **a** Kaplan–Meier curves for abstinence length until first drink during 3 months of acamprosate therapy. **b** *TSPAN5* SNPs were associated with several AUD acamprosate treatment response phenotypes. SNPs within the *TSPAN5* gene (GRCh37/hg19: chr4:99939518–99579812) were tested for association. Single SNPs were evaluated individually as predictors of time until return to alcohol consumption following acamprosate treatment, and time until return to heavy alcohol consumption using multivariable Cox proportional hazard (CPH) models. SNP association with the binary outcome of complete abstinence from any drinking during 3 months of acamprosate therapy was evaluated using multivariable logistic regression models. Models were adjusted for days sober prior to treatment, baseline Penn Alcohol Craving Scale (PACS), and study site. A total of 241 European-American subjects passed quality control (QC) and completed 3 month followup and were therefore included in the logistic outcome models. A total of 305 European-American subjects had at least 1 week of followup time and were included in the CPH analyses of abstinence length until first drink or first heavy drinking day during 3 months of acamprosate therapy. Results of the CPH analyses are displayed using Kaplan-Meier plots, and are not adjusted for multiple testing. Analyses and plots were generated using Rstudio (version 9.4.2). The odds ratios or hazard ratio are represented as HR or OR (95% confidence interval), with a value >1 indicating worse outcome, i.e. variant genotypes for all three SNPs were associated with shorter abstinence length until first drinking or heavy drinking during 3 months of acamprosate treatment. *P* values < 0.05 have been bolded.

We next set out to determine whether SNPs within *TSPAN5* might be associated with the length of abstinence until the first drink of alcohol during 3 months of acamprosate treatment for AUD patients enrolled in the Mayo Clinic Center for the Individualized Treatment of Alcoholism clinical trial (Fig. 5a and Supplementary Table 5) [17, 30]. Clinical characteristics for those subjects are listed in Supplemental Table 6. Specifically, the most significant three *TSPAN5* SNPs (rs11940430, rs4699354, and rs10029405) were in tight linkage disequilibrium (LD) (*R*^*2*^ > 0.98) and were associated with length of abstinence during 3 months of acamprosate treatment. Those same three SNPs were also associated with a different but related clinical phenotype, i.e. abstinence length until heavy drinking or complete abstinence during 3 months of acamprosate treatment (Fig. 5b). In addition, homozygous variant genotypes for those same three SNPs were also associated with higher risk of relapse during 3 months of acamprosate treatment (Fig. 5b). Finally, the three *TSPAN5* SNPs shown in Fig. 5a were also eQTLs for the expression of TSPAN5 in many brain regions (Supplementary Table 7), specifically frontal cortex, cerebellum, medulla and putamen based on data obtained from the 1231 brain tissue samples (up to ten brain regions) that have been archived in the BRAINEAC database (http://www.braineac.org/) which includes 134 brains from individuals free of neurodegenerative disorders. These results, taken together, indicate that genetic variants that are associated with *TSPAN5* expression might be biomarkers for abstinence length in AUD patients treated with acamprosate.

In summary, the results of this series of experiments suggest that TSPAN5 may play a significant role in 5-HT and kynurenine regulation and metabolism. Both of these tryptophan metabolites have been reported to play important roles in neuropsychiatric disorders [18, 31–34]. That may result, in part, from an interaction of TSPAN5 with a series of vesicle-related proteins or its effect on the expression of enzymes involved in monoamine neurotransmitter biosynthesis and metabolism. Furthermore, as a result of the downregulation of TSPAN5 in the presence of either EtOH or acamprosate, downstream genes involved in 5-HT biosynthesis and metabolism, as well as IFN signaling pathways were also down-regulated. Finally, *TSPAN5* SNP genotypes appeared to be associated with acamprosate treatment outcomes in AUD patients—although those observations will require replication. This series of results represent a potentially important step in the process of obtaining functional insight into molecular mechanisms underlying the role of *TSPAN5* in the regulation of two major tryptophan pathway metabolites, 5-HT and kynurenine, as well as individualized treatment outcomes for AUD patients treated with acamprosate.

## Discussion

The present study provides the first evidence that *TSPAN5* genetic variation might be associated with acamprosate treatment outcomes in AUD patients. AUD is the most prevalent substance use disorder [35]. However, only three drugs—acamprosate, naltrexone and disulfiram—have received FDA approval for the treatment of AUD in the United States, and only a small proportion (~35%) of patients respond to treatment with these agents by achieving sustained abstinence [17, 36, 37]. It would represent a major achievement for precision medicine if we were to develop ways to better individualize the drug therapy of AUD patients in order to increase the frequency of the achievement of abstinence and to select the patients most likely to respond prior to the initiation of drug therapy.

Acamprosate, an NMDA glutamate receptor antagonist, is a synthetic compound with a chemical structure similar to those of the neurotransmitter gamma-aminobutyric acid (GABA) and the amino acid taurine [38]. The AUD therapy literature and studies of the mechanism of action of acamprosate have most often focused on its effects on the balance between GABAergic inhibitory and glutamatergic excitatory effects. For example, we reported previously that plasma glutamate concentrations can serve as pharmacometabolomic biomarkers for acamprosate treatment outcomes in AUD patients [39]. However, the present study was designed to explore the biological function of TSPAN5—with a focus on the tryptophan metabolic pathway—by using human iPSC-derived CNS-like cells (i.e. astrocytes and neurons), as well as a microglial cell line as cellular model systems to study molecular and genomic signatures for AUD as well as mechanisms underlying individual variation in response to acamprosate. We observed that acamprosate, an NMDA antagonist, appeared to have “ethanol-like effects” on the expression of genes associated with monoamine neurotransmitter biosynthesis and metabolism as shown in Figs. 1d, g and 2a–d. It is of interest that Krystal and colleagues have reported that “ethanol-like effects” might involve not only serotonergic and noradrenergic mechanisms but also glutamatergic mechanisms [40]. For example, another NMDA antagonist, ketamine, produced ethanol-like effects in a dose-dependent fashion in detoxified AUD patients. In addition, ketamine did not increase craving for ethanol. The mechanism underlying NMDA antagonist-induced ethanol-like effects remains to be determined [41–43].

*TSPAN5* is an alcohol responsive gene that plays a role in the regulation of 5-HT and kynurenine concentrations. Both EtOH and acamprosate decreased TSPAN5 expression, ultimately leading to decreased 5-HT concentrations in cell culture medium. These molecular mechanism(s) could be multifactorial in nature as a result of acamprosate’s effects on neurotransmission, neuroinflammation and/or intracellular signaling in AUD patients. The functional genomic data from our studies of iPSC-derived CNS cells have opened a new avenue for understanding the biological role of TSPAN5 in AUD. We utilized iPSC-derived astrocytes which are expandable to perform a series of functional genomic studies, studies that required a large number of cells. Specifically, we performed gene expression profiling before and after TSPAN5 knockdown in iPSC-derived astrocytes and found that genes associated with changes in expression after the knockdown of TSPAN5 were enriched in IFN signaling pathways. We also determined the possible influence of *TSPAN5* on levels of 5-HT and kynurenine, two major tryptophan metabolites that have been implicated in a variety of neuropsychiatric disorders [18, 31, 32, 34]. Unfortunately, kynurenine concentrations were below the limit of detection in iPSC-derived astrocyte or neuron culture media. These experimental data serve to re-emphasize the importance of the use of iPSC-derived CNS-like cells as tools for the study of neuropsychiatric disorders. The data shown in Fig. 4 represents an example of the use of iPSC-derived CNS-like cells to generate hypotheses and to identify novel biology. Specifically, we performed gene expression profiling before and after knockdown using iPSC-derived astrocytes and found that genes related to interferon signaling pathways displayed significantly altered expression. Those results stimulated us to test a series of genes associated with interferon signaling and to perform interferon-stimulated response element reporter assays. It should be pointed out that iPSC-derived astrocytes are immunocompetent because they can respond to inflammatory stimuli and they can sustain inflammation by producing pro-inflammatory cytokines, similar to the behavior of primary astrocytes [20]. However, microglia are the most prominent immune cells in the CNS. As a result, we replicated these findings using HMC3 cells, as shown in Supplementary Fig. 3. Strikingly, TSPAN5 knockout in HMC3 cells was associated with the downregulation of kynurenine and ISRE luciferase activities, results compatible with observations that we made when the cells were treated with either EtOH or acamprosate. We observed that both EtOH and acamprosate behaved in a similar fashion in this system.

It should be pointed out that the concentrations of acamprosate used in our cell culture studies were within the range of acamprosate concentrations observed in AUD patients treated with acamprosate [21]. The cells were also exposed to EtOH at 5–50 mM, concentrations that are considered physiologically relevant. It is currently accepted that treatment with EtOH for 24 hours is considered as an acute exposure [44]. However, the effects of alcohol vary based on the length of exposure and the EtOH concentration. As a result, molecular mechanisms related to the diverse effects of chronic and acute EtOH exposure remain unclear. For example, our results suggest that decreased TSPAN5 mRNA expression after EtOH treatment for 24 h leads to the downregulation of genes involved in IFN signaling pathways. However, it is well-documented that chronic EtOH exposure can severely damage multiple organs including the liver and brain through the activation of immune-related pathways including IFN signaling pathways [45, 46]. As a result, acute EtOH exposure might have anti-inflammatory effects while chronic EtOH exposure could potentially switch anti-inflammatory to pro-inflammatory responses [46].

The present study used human iPSC-derived CNS-like cells to perform functional genomic studies. Obviously, iPSC-derived cell lines, like any cell lines, have limitations. For example, it should be emphasized that iPSC-derived CNS cells are “region-specific”. The present study utilized forebrain-specific glial and neural cells, cells that have been implicated in the pathophysiology of AUD [47, 48]. However, future studies that include different brain regions will be required to pursue the results reported here as well as the application of co-culture systems or of iPSC-derived organoids in an attempt to mimic glial-neuronal cell communications in vitro.

In summary, the present study has highlighted the fact that TSPAN5 can regulate the two major metabolites of the tryptophan metabolic pathway as well as CNS immune responses. Specifically, genes that were modulated by *TSPAN5* were enriched in IFN signaling pathways. Our data also raise the possibility that *TSPAN5*, which appears to play a functional role in CNS immune response, might also contribute to individualized acamprosate treatment outcomes through a novel pharmacogenomic mechanism.

## Supplementary information

Supplementary Text

Supplementary Figure and Table legends

Supplementary Figures

Supplementary Tables

## References

[1] Gupta M, Neavin D, Liu D, Biernacka J, Hall-Flavin D, Bobo WV (2016). TSPAN5, ERICH3 and selective serotonin reuptake inhibitors in major depressive disorder: pharmacometabolomics-informed pharmacogenomics. Mol Psychiatry.

[2] Kranzler HR, Zhou H, Kember RL, Vickers Smith R, Justice AC, Damrauer S (2019). Genome-wide association study of alcohol consumption and use disorder in 274,424 individuals from multiple populations. Nat Commun.

[3] Walters RK, Polimanti R, Johnson EC, McClintick JN, Adams MJ, Adkins AE (2018). Transancestral GWAS of alcohol dependence reveals common genetic underpinnings with psychiatric disorders. Nat Neurosci.

[4] Gelernter J, Sun N, Polimanti R, Pietrzak RH, Levey DF, Lu Q (2019). Genome-wide association study of maximum habitual alcohol intake in >140,000 U.S. European and African American veterans yields novel risk loci. Biol Psychiatry.

[5] Clarke TK, Adams MJ, Davies G, Howard DM, Hall LS, Padmanabhan S (2017). Genome-wide association study of alcohol consumption and genetic overlap with other health-related traits in UK Biobank (N=112 117). Mol Psychiatry.

[6] Ho M-F, Zhang L, Moon I, Skime M, Ho AM-C, Choi D-S (2019). TSPAN5, an alcohol responsive gene that alters serotonin levels in human induced pluripotent stem cells: novel molecular links to alcohol use disorder. Alcohol: Clin Exp Res.

[7] Shen WW (2018). Anticraving therapy for alcohol use disorder: a clinical review. Neuropsychopharmacol Rep.

[8] Kranzler HR, Edenberg HJ (2010). Pharmacogenetics of alcohol and alcohol dependence treatment. Curr Pharm Des.

[9] Litten RZ, Bradley AM, Moss HB (2010). Alcohol biomarkers in applied settings: recent advances and future research opportunities. Alcohol: Clin Exp Res.

[10] Heilig M, Goldman D, Berrettini W, O’Brien CP (2011). Pharmacogenetic approaches to the treatment of alcohol addiction. Nat Rev Neurosci.

[11] Berditchevski F, Odintsova E (2007). Tetraspanins as regulators of protein trafficking. Traffic.

[12] Lonsdale J, Thomas J, Salvatore M, Phillips R, Lo E, Shad S (2013). The Genotype-Tissue Expression (GTEx) project. Nat Genet.

[13] Kane CJM, Drew PD (2016). Inflammatory responses to alcohol in the CNS: nuclear receptors as potential therapeutics for alcohol-induced neuropathologies. J Leukoc Biol.

[14] Anton RF, O’Malley SS, Ciraulo DA (2006). Combined pharmacotherapies and behavioral interventions for alcohol dependence: the combine study: a randomized controlled trial. JAMA.

[15] Pelc I, Verbanck P, Bon OL, Gavrilovic M, Lion K, Lehert P (1997). Efficacy and safety of acamprosate in the treatment of detoxified alcohol-dependent patients: a 90-day placebo-controlled dose-finding study. Br J Psychiatry.

[16] Whitworth AB, Oberbauer H, Fleischhacker WW, Lesch OM, Walter H, Nimmerrichter A (1996). Comparison of acamprosate and placebo in long-term treatment of alcohol dependence. Lancet.

[17] Karpyak VM, Biernacka JM, Geske JR, Jenkins GD, Cunningham JM, Rüegg J (2014). Genetic markers associated with abstinence length in alcohol-dependent subjects treated with acamprosate. Transl Psychiatry.

[18] Vadodaria KC, Ji Y, Skime M, Paquola AC, Nelson T, Hall-Flavin D (2019). Altered serotonergic circuitry in SSRI-resistant major depressive disorder patient-derived neurons. Mol Psychiatry.

[19] Vadodaria KC, Ji Y, Skime M, Paquola A, Nelson T, Hall-Flavin D (2019). Serotonin-induced hyperactivity in SSRI-resistant major depressive disorder patient-derived neurons. Mol Psychiatry.

[20] Wen Z, Nguyen HN, Guo Z, Lalli MA, Wang X, Su Y (2014). Synaptic dysregulation in a human iPS cell model of mental disorders. Nature.

[21] Mason BJ, Goodman AM, Dixon RM, Hameed MHA, Hulot T, Wesnes K (2002). A pharmacokinetic and pharmacodynamic drug interaction study of acamprosate and naltrexone. Neuropsychopharmacology.

[22] Dobin A, Davis CA, Schlesinger F, Drenkow J, Zaleski C, Jha S (2013). STAR: ultrafast universal RNA-seq aligner. Bioinforma (Oxf, Engl).

[23] Langmead B, Salzberg SL (2012). Fast gapped-read alignment with Bowtie 2. Nat Meth.

[24] Liao Y, Smyth GK, Shi W (2013). The Subread aligner: fast, accurate and scalable read mapping by seed-and-vote. Nucleic Acids Res.

[25] Love MI, Huber W, Anders S (2014). Moderated estimation of fold change and dispersion for RNA-seq data with DESeq2. Genome Biol.

[26] Subramanian A, Tamayo P, Mootha VK, Mukherjee S, Ebert BL, Gillette MA (2005). Gene set enrichment analysis: A knowledge-based approach for interpreting genome-wide expression profiles. Proc Natl Acad Sci.

[27] Mootha VK, Lindgren CM, Eriksson K-F, Subramanian A, Sihag S, Lehar J (2003). PGC-1α-responsive genes involved in oxidative phosphorylation are coordinately downregulated in human diabetes. Nat Genet.

[28] Lira MC, Sarda V, Heeren TC, Miller M, Naimi TS (2020). Alcohol policies and motor vehicle crash deaths involving blood alcohol concentrations below 0.08. Am J Preventive Med.

[29] Hanisch U-K, Kettenmann H (2007). Microglia: active sensor and versatile effector cells in the normal and pathologic brain. Nat Neurosci.

[30] Karpyak VM, Geske JR, Hall-Flavin DK, Loukianova LL, Schneekloth TD, Skime MK (2019). Sex-specific association of depressive disorder and transient emotional states with alcohol consumption in male and female alcoholics. Drug Alcohol Depend.

[31] Liu D, Ray B, Neavin DR, Zhang J, Athreya AP, Biernacka JM (2018). Beta-defensin 1, aryl hydrocarbon receptor and plasma kynurenine in major depressive disorder: metabolomics-informed genomics. Transl Psychiatry.

[32] Kindler J, Lim CK, Weickert CS, Boerrigter D, Galletly C, Liu D, et al. Dysregulation of kynurenine metabolism is related to proinflammatory cytokines, attention, and prefrontal cortex volume in schizophrenia. Mol Psychiatry. 2019. 10.1038/s41380-019-0401-9. 10.1038/s41380-019-0401-9 PMC7577855 30940904

[33] Allen AP, Naughton M, Dowling J, Walsh A, O’Shea R, Shorten G (2018). Kynurenine pathway metabolism and the neurobiology of treatment-resistant depression: Comparison of multiple ketamine infusions and electroconvulsive therapy. J Psychiatr Res.

[34] Wu H, Denna TH, Storkersen JN, Gerriets VA (2019). Beyond a neurotransmitter: the role of serotonin in inflammation and immunity. Pharmacol Res.

[35] Robinson SM, Adinoff B (2016). The classification of substance use disorders: historical, contextual, and conceptual considerations. Behav Sci (Basel).

[36] Mann K, Lehert P, Morgan MY (2004). The efficacy of acamprosate in the maintenance of abstinence in alcohol-dependent individuals: results of a meta-analysis. Alcohol: Clin Exp Res.

[37] Boothby LA, Doering PL (2005). Acamprosate for the treatment of alcohol dependence. Clin Therapeutics.

[38] Mason BJ, Heyser CJ (2010). Acamprosate: a prototypic neuromodulator in the treatment of alcohol dependence. CNS Neurol Disord Drug Targets.

[39] Nam HW, Karpyak VM, Hinton DJ, Geske JR, Ho AMC, Prieto ML (2015). Elevated baseline serum glutamate as a pharmacometabolomic biomarker for acamprosate treatment outcome in alcohol-dependent subjects. Transl Psychiatry.

[40] Krystal JH, Webb E, Cooney N, Kranzler HR, Charney DS (1994). Specificity of ethanollike effects elicited by serotonergic and noradrenergic mechanisms. Arch Gen Psychiatry.

[41] Krystal JH, Petrakis IL, Krupitsky E, SchÜtz C, Trevisan L, D’souza DC (2003). NMDA Receptor antagonism and the ethanol intoxication signal. Ann N. Y Acad Sci.

[42] Krystal JH, Petrakis IL, Mason G, Trevisan L, D’Souza DC (2003). N-methyl-d-aspartate glutamate receptors and alcoholism: reward, dependence, treatment, and vulnerability. Pharmacol Therapeutics.

[43] Krystal JH, Petrakis IL, Webb E, Cooney NL, Karper LP, Namanworth S (1998). Dose-related ethanol-like effects of the NMDA antagonist, ketamine, in recently detoxified alcoholics. Arch Gen Psychiatry.

[44] Dolganiuc A, Szabo G (2009). In vitro and in vivo models of acute alcohol exposure. World J Gastroenterol.

[45] Nagy LE (2015). The role of innate immunity in alcoholic liver disease. Alcohol Res.

[46] Mandrekar P, Bala S, Catalano D, Kodys K, Szabo G (2009). The Opposite effects of acute and chronic alcohol on lipopolysaccharide-induced inflammation are linked to IRAK-M in human monocytes. J Immunol.

[47] Swift RM, Aston ER (2015). Pharmacotherapy for alcohol use disorder: current and emerging therapies. Harv Rev Psychiatry.

[48] Bradshaw SD, Shumway ST, Dsauza CM, Morris N, Hayes ND (2017). Hope, coping skills, and the prefrontal cortex in alcohol use disorder recovery. Am J Drug Alcohol Abus.

