# Supplementary material for: TSPAN5 influences serotonin and kynurenine: pharmacogenomic mechanisms related to alcohol use disorder and acamprosate treatment response
Source: Mol Psychiatry. 2020 Aug 4;26(7):3122–33. doi: 10.1038/s41380-020-0855-9 (PMC7858703; doi:10.1038/s41380-020-0855-9)
Supplement: Supplementary file 1 — Supplementary Text [file 41380_2020_855_MOESM1_ESM.docx]

**Supplementary text**

**TSPAN5 influences serotonin and kynurenine: pharmacogenomic mechanisms related to alcohol use disorder and acamprosate treatment response**

# Ming-Fen Ho, Ph.D.^1^, Cheng Zhang, Ph.D.^1^, Lingxin Zhang, Ph.D.^1^, Lixuan Wei, M.D. Ph.D.^1^, Ying Zhou, Ph.D.^2^, Irene Moon^1^, Jennifer R. Geske, M.S.^3^, Doo-Sup Choi, Ph.D.^1^, Joanna Biernacka, Ph.D.^3^, Mark Frye, M.D.^4^, Zhexing Wen, Ph.D.2, Victor M Karpyak, M.D., Ph.D.^4^ , Hu Li, Ph.D.^1^, and Richard Weinshilboum, M.D.^1^.^*^

***Corresponding author:** Richard Weinshilboum, M.D., Mayo Clinic 200 First Street SW, Rochester, MN 55905, Phone: 507-284-2790, FAX: 507-284-4455, Email: [weinshilboum.richard@mayo.edu](mailto:weinshilboum.richard@mayo.edu)

**SUPPLEMENTARY Methods and materials**

**Subjects**

The Mayo Clinic Center for the Individualized Treatment of Alcoholism recruited 442 AUD subjects. Clinical data and DNA samples were obtained at baseline and clinical data and an additional blood sample were obtained after 3 months of acamprosate treatment. The retention rate for the study was 60.4% with 81% medication compliance (defined as more than 50% of the pills taken) and an abstinence rate of 58.9% at 3 months. Specifically, 305 European American subjects had acamprosate treatment outcomes available, i.e. abstinence length during acamprosate therapy (1). In addition, induced pluripotent stem cells (iPSCs) were generated from five healthy subjects from Mayo Biobank. Confidentiality was maintained for all study participants. This study was conducted in accordance with protocols reviewed and approved by the Mayo Clinic Institutional Review Board (reference number: 10-006845). All subjects whose skin biopsy samples were used to generate iPSCs in this study gave their consent for participation in the study and for publication of the study results in peer-reviewed scientific journals.

**Generation of patient-derived iPSCs, and glial and neuronal cell differentiation**

Fibroblasts from skin biopsies for all subjects were utilized for iPSC reprogramming using the CytoTune™-iPS 2.0 Sendai Reprogramming Kit (A16517, Thermo fisher, USA). Patient-derived iPSC were characterized as described previously (2, 3). The iPSCs were then differentiated into astrocytes and forebrain neurons as previously described (4). Briefly, iPS cells were cultured on Matrigel with mTeSR1 media (STEMCELL technology, MA, USA). iPS cell colonies were detached from the Matrigel (Corning) with 1 mg ml^−1^ collagenase treatment for 1 hour and the cells were suspended in embryoid body (EB) medium, consisting of FGF-2-free iPS cell medium supplemented with 2 μM dorsomorphin (sigma) and 2 μM A-83 (sigma), in non-treated polystyrene plates for 6 days with a daily medium change (to renew 50% of the medium). After 6 days, EB medium was replaced by neural induction medium (hNPC medium) consisting of DMEM/F12, N2 supplement, NEAA, 2 ug ml^−1^ heparin (Tocris Bioscience) and 2 μM cyclopamine (Tocris Bioscience). The floating EBs were then transferred to Matrigel-coated 6-well plates at day 7 to form neural tube-like rosettes. The attached rosettes were kept for 15 days with hNPC medium change every other day. On day 22, the rosettes were picked mechanically and transferred to low attachment plates in hNPC medium containing B27 (Thermo fisher). After two days in culture, for astrocyte differentiation, resuspended neural progenitor spheres were dissociated with Accutase (STEMCELL technology, MA, USA) and placed onto Matrigel-coated plates in astrocyte culture medium (1801, ScienCell). However, for neuronal differentiation, cells were placed onto Matrigel-coated plates in neuronal culture medium, consisting of Neurobasal medium supplemented with 2 mM L-glutamax, B27, 10 ng ml^−1^ BDNF and 10 ng ml^−1^ GDNF. Medium (50%) was replaced once a week during continuous culture (4). The NPCs were cultured for 15 days to form neural rosette structures, and these NPCs expressed nestin, PAX6, EMX1, FOXG1 and OTX2. The iPSC-derived forebrain neurons expressed several cortical layer markers, including TBR1, CTIP2, BRN2 and SATB2. 90% of the iPSC-derived forebrain neurons are glutamatergic excitatory neurons which express VGLUT1 and α-CAMKII. The iPSC-derived forebrain neurons are functionally mature 4 weeks after differentiation from hNPCs. Specifically, they form synapses, fire action potentials, and have spontaneous synaptic activity (4).

**Cell culture and drug treatment**

HMC3 cells (ATCC CRL-3304, Manassas, VA, USA) were cultured in EMEM media (Cellgro, Manassas, VA, USA) supplemented with 10% FBS (Atlanta Biologicals, Flowery Branch, GA, USA). Cells were then treated with various concentrations of ethanol (EtOH) or acamprosate for 24 hours within the range of concentrations observed during acamprosate treatment of patients with AUD (5).

**RNA Sequencing and data analysis**

Total RNA was extracted using the RNeasy mini kit (Qiagen, Valencia, CA, USA). We used iPSC-derived astrocytes from one individual with two biological replicates to perform mRNA expression profiling before and after TSPAN5 knockdown. RNA-seq findings were validated using four additional human iPSC-derived astrocytes. RNA-seq experiments were conducted by GENEWIZ using an Illumina HiSeq 4000 with eight samples in each lane using 100bp paired end index reads. Fastq files containing paired RNASeq reads were aligned with STAR (6) against the UCSC human reference genome (hg19) using Bowtie 2.2.3 with default settings (7). Gene level counts from uniquely mapped, non-discordant read pairs were obtained using the subRead featureCounts program (v1.4.6) (8) and gene models from the UCSC hg19 Illumina iGenomes annotation package. Differential expression analysis was also performed using the DESeq2 package with default parameters (9). Gene set enrichment analysis (GSEA) software was used to perform pathway analysis (10, 11).

**Real time PCR**

The PCR reactions contained 200 ng of total RNA, 5 µl of 2X SYBR green qPCR master mix (Life technologies, CA USA), 1 µl of gene specific primer and distilled water up to 10 µl final volume per reaction. Primer sets for real time PCR are listed in **Supplementary Table 1**. Real time PCR reactions were performed in duplicate using the Applied Biosystems ViiA 7™ real-Time PCR System (Life Technologies, Carlsbad, CA, USA). The 2^-ΔΔCt^ method was employed for statistical data analysis.

**High-performance liquid chromatography (HPLC)**

Cells were seeded in 6-well plates at a density of 2.5 × 10^5^ cells per well in 2 ml culture medium. Serotonin (5HT) in the culture medium was separated and detected using electrochemical detection. The samples from culture medium were collected and centrifuged at 6000 g for 5 min at 4°C to remove any cells or cellular debris. Culture medium (50 μl) was used to analyze samples for catecholamines and serotonin on a Shimadzu HPLC System (Shimadzu Corporation, Kyoto, Japan), equipped with a CBM-20A controller, LC-20AD pump and with UltiMate™ 3000 ECD-3000RS Electrochemical Detector (Thermo Scientific, San Jose, CA). Chromatographic separation was achieved on a Shimadzu C_18_ reversed-phase column 150 × 4.6 mm, 3 μm particle size (Shimadzu Corporation, Kyoto, Japan) coupled with a Phenomenex Security Guard™ C18 guard column 4 × 3 mm (Phenomenex ,Torrance, CA). The mobile phase was degassed as well as vacuum filtered through 0.22 μm nylon membranes. The system was equilibrated with mobile phase MDTM (Thermo Scientific, San Jose, CA), through which mobile phase was pumped at a rate of 1.0 mL/min, with the oven held at 40⁰C and an analyze time of 10 mins. Detection involved an electrode with a two-channel coulometric cell (6011RS, Thermo Scientific, San Jose, CA). The cell potential was set at E1 = -175 mV (100 nA), E2 = +475 mV (1 µA). Data were acquired and processed with Chromeleon software version 7.2.8 (Thermo Scientific, San Jose, CA). Peaks from samples were then identified and quantified by comparison with catecholamine (Thermo Scientific, San Jose, CA) and serotonin (Sigma, St. Louis, MO) standards. In addition, kynurenine (KYN) in the culture medium was separated and detected using a HPLC-fluorescence detection method.  Samples were assayed on a Shimadzu HPLC System (Shimadzu Corporation, Kyoto, Japan), equipped with a CBM-20A controller, two LC-20AD pumps (Shimadzu Corporation, Kyoto, Japan). Chromatographic separation of kynurenine was achieved on a LiChrosorb® RP 18-5 (Supelco, Bellefonte, PA) C_18_ reversed-phase column (250 mm × 4.6 mm, 5 μm) and separation of tryptophan was accomplished using a Shimadzu C_18_reversed-phase column (150 × 4.6 mm, 3 μm) (Shimadzu Corporation, Kyoto, Japan). The mobile phase consisted of potassium dihydrogen phosphate-disodium hydrogen phosphate (final 40.78-25.87 mmol/L, pH 7.0, 25⁰C) and methanol (gradient from 75:25 to 20:80, v/v, 20 mins) at a flow rate of 0.5 mL/min at 25 °C coupled with a Phenomenex Security Guard™ C_18_ guard column 4 × 3 mm (Phenomenex, Torrance, CA). The mobile phase was degassed as well as vacuum filtered through 0.22 μm nylon membranes before use. The HPLC system was equipped with an RF-20A XS fluorescence detector (Shimadzu Corporation, Kyoto, Japan). Culture supernatant (50 μl) was used to analyze kynurenine by fluorescence detection with an excitation wavelength of 365 nm and an emission wavelength of 480 nm; TRP was detected with excitation at 225 nm, emission at 365 nm and an injected volume of 10 μl. The HPLC was also equipped with a diode array detector SPD-M20A (Shimadzu Corporation, Kyoto, Japan) for UV detection. The identity of KYN was confirmed by comparing UV spectra with standards. KYN showed UV absorption at 280 nm and 360 nm. Data were acquired and processed with LabSolutions software version 5.85(Shimadzu Corporation, Kyoto, Japan). Peaks from samples were then identified and quantified by comparison with KYN standards (Sigma, St. Louis, MI).

**Immunofluorescence staining and confocal imaging analysis**

# iPSC-derived astrocytes and neurons were grown on glass coverslips for 24 hours. Cells were then fixed in 4% paraformaldehyde at room temperature for 15 min. The cells were washed in cold PBS and permeablized with 0.2% Triton X-100 in PBS. After blocking for one hour with 3% BSA, cells were incubated with primary antibody (see Supplementary Table 1) overnight at 4°C. The secondary antibody was used at a 1:1000 dilution for an hour. VECTASHIELD antifade mounting media with DAPI (VECTOR laboratory, Burlingame, CA, USA) was used to stain the cell nuclei (blue). Slides were visualized using fluorescence microscopy (Olympus, FV1200).

**Immunoprecipitation (IP), mass spectrometry and Western blot analysis**

iPSC-derived astrocytes (1x10^7^) were resuspended in 1 ml IP lysis buffer containing 5 µl protease inhibitor cocktail (Qiagen, Valencia, CA, USA) and were incubated on ice for 30 min. Cells were then centrifuged at 12,000 g at 4°C for 15 min. and supernatant was collected. Protein A agarose (ThermoScientific, Madison, WI, USA) was prepared and washed with IP lysis buffer. A pre-cleaning step was performed in order to clean the background. Cell lysates containing protein A agarose beads were rotated at 4°C for an hour. Supernatant was collected after centrifugation. At that point, input (50 µl) was collected and stored at -80°C. Anti-TSPAN5 (1:50) antibody (MyBioSource: MBS1498957, San Diego, CA, USA) was used to perform IP. IgG (Cell Signaling Technology, Danvers, MA, USA), was used as a negative control. Specifically, IP samples containing protein A agarose beads were rotated at 4°C overnight. Immunoprecipitates were washed three times with ice cold lysis buffer, and proteins were eluted with 50µl 1X Laemmli loading buffer. Proteins pulled down by anti-TSPAN5 antibody were separated on 4-12% SDS-PAGE gels for mass spectrometry (The Taplin Biological Mass Spectrometry Facility, Boston, MA, USA). In some experiments, proteins were transferred onto PDVF membranes. After blocking, membranes were incubated with primary antibody against TSPAN5 at 4°C overnight. The washed membranes were then incubated with secondary antibody (1:15000 dilution) for an hour at room temperature. The membrane was visualized using super signal ECL substrate (Thermo Scientific, Madison, WI, USA).

**TSPAN5 siRNA knockdown and CRISPR/cas9 knockout studies**

# Smart pooled TSPAN5 siRNA (M-010634-02) and negative control were purchased from Dharmacon (Chicago, IL, USA). siRNA was transfected into the cells by lipofectamine RNAimax reagent (Life Technologies, Carlsbad, CA, USA). Briefly, cells were seeded in a six- well plate (1.5x10^6^ cells/plate) and the transfection reaction contained 2.5x10^5^ cells/well, 250 µl of opti-MEM medium, 7 µl of lipofectamine RNAimax reagent and 500 nM of siRNA. TSPAN5 Double Nickase Plasmid that contained two TSPAN5 specific guide RNA sequences located in exon 1: TTGGCTTCAATGTCATATTT and TTGATGCAACAACTGACTTC (sc-404621-NIC, Santa Cruz Biotechnology, Dallas, Texas, USA) was transfected into HMC3 cells and the cells recovered for 24 hours in EMEM medium supplemented with 10% FBS. Transfected cells were cultured in medium containing 0.2μg/ml puromycin for 10 days for selection. Cells were seeded on 96 well culture plates using EMEM medium supplemented with 10% FBS for single clone selection. TSPAN5 expression was quantified by Western blot analysis. Rabbit anti-Human TSPAN5 polyclonal antibody was purchased from Mybiosource (catalog number: MBS1498957) (Immunogen:114-232 amino acid spanning exon 4-7). TSPAN5 expression was quantified and normalized to the beta-actin signal by Western blot analysis. Cells exhibiting <10% normalized TSPAN5 signal were considered to have TSPAN5 knocked out.

**Reporter gene assay**

HMC3 cells or iPSC-derived astrocytes (3 × 10^5^ cells/well) were seeded in 6-well plates the day before transfection. Cells were transfected with 750 ng of firefly luciferase ISRE reporter plasmid and the Renilla luciferase reporter gene plasmid (Promega, WI, USA). The next day, the culture medium was renewed and the cells were treated with either EtOH or acamprosate for an additional 24 hours. Cells were lysed in passive lysis buffer at room temperature for 15 minutes. Firefly and Renilla luciferase activities were determined using the Dual-Luciferase Reporter 1000 Assay System (Promega, Madison, WI, USA). Normalized luciferase activity was calculated for each sample by dividing the firefly luciferase activity by the Renilla luciferase activity as a control to correct for possible variation in transfection efficiency. All experiments were repeated three times in triplicate.

**Statistics**

Gene expression data are presented as mean ± S.E.M. Realtime PCR and luciferase reporter assay results were analyzed using a paired *t* test or ANOVA, followed by Tukey’s multiple comparison test for individual comparisons when significant effects were detected. Differences were considered significant at p<0.05. GraphPad Prism Software v7 (San Diego, CA, USA) was used for data analysis.

**Genotyping and data analysis**

Samples were first genotyped using Illumina HumanCore arrays at the Medical Genome Facility at Mayo Clinic, and subsequently were re-genotyped using Infinium OmniExpressExome-8 BeadChips at NIAAA. Data from the two arrays were quality-controlled, combined and checked for concordance, and additional quality control was performed on the combined dataset. Samples were excluded from analysis if they had a low call rate, extreme heterozygosity, or disagreement between reported sex and genetically determined sex. Sample relatedness was checked by pairwise identical-by-descent (IBD) estimation. SNPs were excluded from analysis if they had a low call rate, a low minor allele frequency, or deviated significantly from Hardy Weinberg Equilibrium. Imputation was conducted using the Michigan Imputation Server with the HRC reference panel (version HRC.r1-1.GRCh37.wgs.mac5.sites). 473 SNPs within *TSPAN5* gene (GRCh37/hg19: chr4:99939518-99579812) gene were tested for association. Single SNPs were evaluated individually as predictors of time until return to alcohol consumption following acamprosate treatment, and time until return to heavy alcohol consumption using multivariable Cox proportional hazard (CPH) models. SNP association with the binary outcome of complete abstinence from any drinking during 3 months of acamprosate therapy was evaluated using multivariable logistic regression models. Models were adjusted for days sober prior to treatment, baseline Penn Alcohol Craving Scale (PACS), and study site. A total of 241 European American subjects passed quality control (QC) and completed 3 month follow up and were therefore included in the logistic outcome models. A total of 305 European American subjects had at least 1 week of follow up time and were included in the CPH analyses of abstinence length until first drink or first heavy drinking day during 3 months of acamprosate therapy. Results of the CPH analyses are displayed using Kaplan-Meier plots, and are not adjusted for multiple testing. Analyses and plots were generated using Rstudio (version 9.4.2).

**References:**

1. Karpyak VM, Biernacka JM, Geske JR, Jenkins GD, Cunningham JM, Rüegg J, et al. Genetic markers associated with abstinence length in alcohol-dependent subjects treated with acamprosate. Translational Psychiatry. 2014;4:e453.

2. Vadodaria KC, Ji Y, Skime M, Paquola AC, Nelson T, Hall-Flavin D, et al. Altered serotonergic circuitry in SSRI-resistant major depressive disorder patient-derived neurons. Molecular Psychiatry. 2019;24(6):808-18.

3. Vadodaria KC, Ji Y, Skime M, Paquola A, Nelson T, Hall-Flavin D, et al. Serotonin-induced hyperactivity in SSRI-resistant major depressive disorder patient-derived neurons. Molecular Psychiatry. 2019;24(6):795-807.

4. Wen Z, Nguyen HN, Guo Z, Lalli MA, Wang X, Su Y, et al. Synaptic dysregulation in a human iPS cell model of mental disorders. Nature. 2014;515(7527):414-8.

5. Mason BJ, Goodman AM, Dixon RM, Hameed MHA, Hulot T, Wesnes K, et al. A Pharmacokinetic and Pharmacodynamic Drug Interaction Study of Acamprosate and Naltrexone. Neuropsychopharmacology. 2002;27:596.

6. Dobin A, Davis CA, Schlesinger F, Drenkow J, Zaleski C, Jha S, et al. STAR: ultrafast universal RNA-seq aligner. Bioinformatics (Oxford, England). 2013;29(1):15-21.

7. Langmead B, Salzberg SL. Fast gapped-read alignment with Bowtie 2. Nat Meth. 2012;9(4):357-9.

8. Liao Y, Smyth GK, Shi W. The Subread aligner: fast, accurate and scalable read mapping by seed-and-vote. Nucleic Acids Research. 2013;41(10):e108-e.

9. Love MI, Huber W, Anders S. Moderated estimation of fold change and dispersion for RNA-seq data with DESeq2. Genome Biology. 2014;15(12):550.

10. Subramanian A, Tamayo P, Mootha VK, Mukherjee S, Ebert BL, Gillette MA, et al. Gene set enrichment analysis: A knowledge-based approach for interpreting genome-wide expression profiles. Proceedings of the National Academy of Sciences. 2005;102(43):15545-50.

11. Mootha VK, Lindgren CM, Eriksson K-F, Subramanian A, Sihag S, Lehar J, et al. PGC-1α-responsive genes involved in oxidative phosphorylation are coordinately downregulated in human diabetes. Nature genetics. 2003;34:267.
