# Supplementary material for: TSPAN5 influences serotonin and kynurenine: pharmacogenomic mechanisms related to alcohol use disorder and acamprosate treatment response
Source: Mol Psychiatry. 2020 Aug 4;26(7):3122–33. doi: 10.1038/s41380-020-0855-9 (PMC7858703; doi:10.1038/s41380-020-0855-9)
Supplement: Supplementary file 2 — Supplementary Figure and Table legends [file 41380_2020_855_MOESM2_ESM.docx]

**Supplementary Figure 1.** (**a**) Time course analysis of 5-HT concentrations in SK-N-BE2 cell culture medium in response to EtOH treatment. (**b**) Dose response curve of 5-HT concentrations in SK-N-BE2 cell culture medium in response to acamprosate treatment.

**Supplementary Figure 2.** iPSC generation, culture and characterization. (**a)** Schematic diagram depicting the timeline of glial and neuronal cell differentiation processes. (**b**) Immunofluorescence staining of iPSC lines, astrocytes and neurons.

**Supplementary Figure 3.** TSPAN5 function in HMC3 cells. **(a)** Lower ISRE luciferase activities were observed in *TSPAN5* knockout cells. (**b**) Knockdown of TSPAN5 resulted in the downregulation of a series of genes involved in interferon signaling, compatible with the data shown in panels (**c)** and (**d)** when the cells were exposed to either EtOH or acamprosate.

**Supplementary Table 1.**  Primer sets and antibodies used in these studies.

**Supplementary Table 2.** TSPAN5 partner proteins in iPSC-derived astrocytes as determined by “pull-down” followed by mass spectrometrometric analysis.

**Supplementary Table 3**. Genes with altered expression after TSPAN5 was knocked down in iPSC-derived astrocytes as determined by RNA-seq (FDR<0.05).

**Supplementary Table 4.** Pathway analysis of genes with altered expression as determined by RNA-seq. after TSPAN5 had been knocked down in iPSC-derived astrocytes.

**Supplementary Table 5.** *TSPAN5* SNPs associated with the length of abstinence during 3 months of acamprosate treatment.

**Supplementary Table 6.** Subject characteristics for subjects enrolled in the Mayo acamprosate clinical trial.

**Supplementary Table 7**. SNPs (rs11940430, rs4699354 and rs10029405) were eQTL for TSPAN5. These SNPs were also associated with the length of abstinence during 3 months of acamprosate treatment. eQTL data extracted from BRAINEAC database (http://www.braineac.org/). P values <0.05 were highlighted.
