# Supplementary material for: TSPAN5 influences serotonin and kynurenine: pharmacogenomic mechanisms related to alcohol use disorder and acamprosate treatment response
Source: Mol Psychiatry. 2020 Aug 4;26(7):3122–33. doi: 10.1038/s41380-020-0855-9 (PMC7858703; doi:10.1038/s41380-020-0855-9)
Supplement: Supplementary file 3 — Supplementary Figures [file 41380_2020_855_MOESM3_ESM.pptx]

## Slide 1
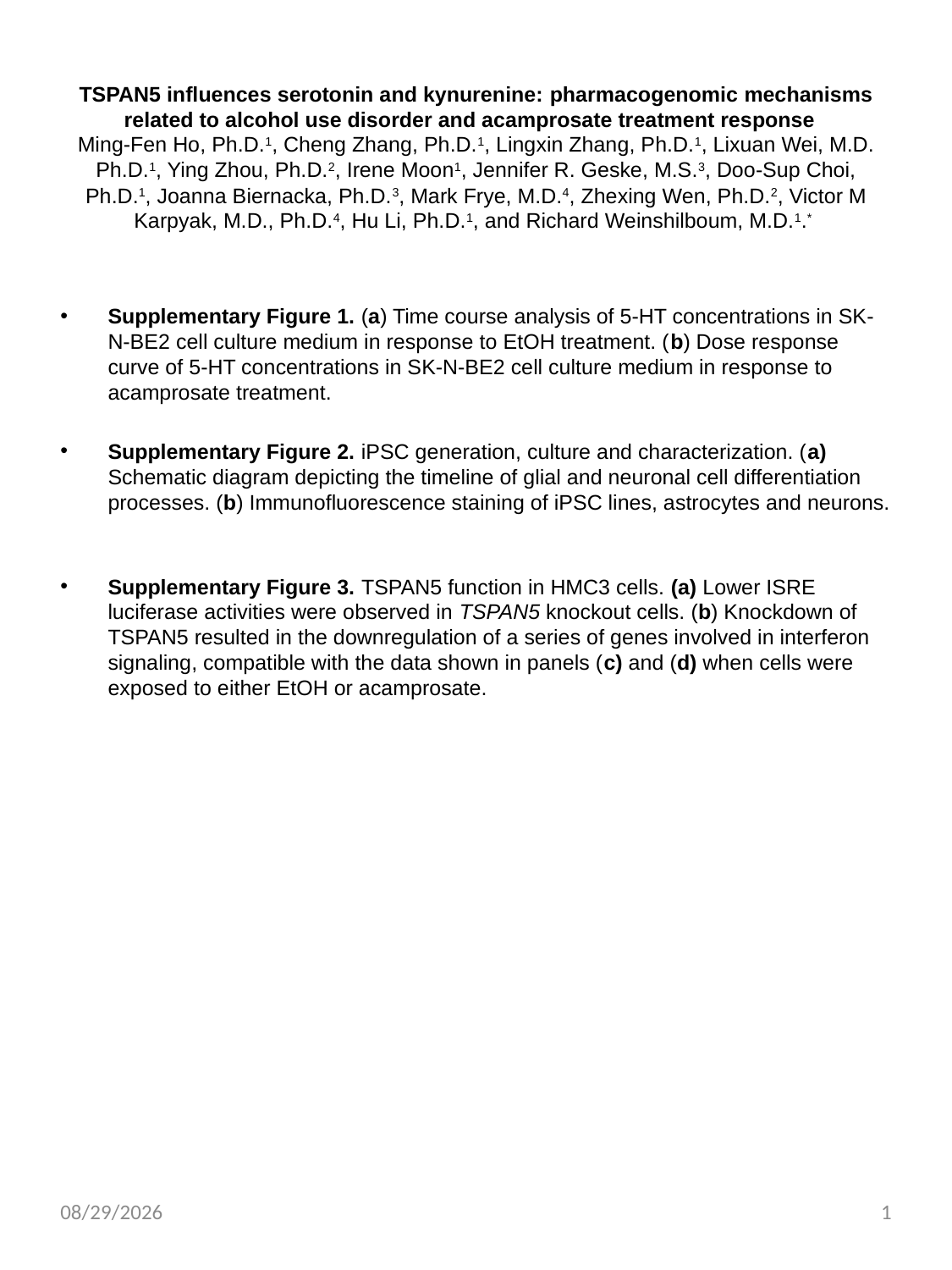

# TSPAN5 influences serotonin and kynurenine: pharmacogenomic mechanisms related to alcohol use disorder and acamprosate treatment response Ming-Fen Ho, Ph.D.1, Cheng Zhang, Ph.D.1, Lingxin Zhang, Ph.D.1, Lixuan Wei, M.D. Ph.D.1, Ying Zhou, Ph.D.2, Irene Moon1, Jennifer R. Geske, M.S.3, Doo-Sup Choi, Ph.D.1, Joanna Biernacka, Ph.D.3, Mark Frye, M.D.4, Zhexing Wen, Ph.D.2, Victor M Karpyak, M.D., Ph.D.4, Hu Li, Ph.D.1, and Richard Weinshilboum, M.D.1.*
Supplementary Figure 1. (a) Time course analysis of 5-HT concentrations in SK-N-BE2 cell culture medium in response to EtOH treatment. (b) Dose response curve of 5-HT concentrations in SK-N-BE2 cell culture medium in response to acamprosate treatment.
Supplementary Figure 2. iPSC generation, culture and characterization. (a) Schematic diagram depicting the timeline of glial and neuronal cell differentiation processes. (b) Immunofluorescence staining of iPSC lines, astrocytes and neurons.
Supplementary Figure 3. TSPAN5 function in HMC3 cells. (a) Lower ISRE luciferase activities were observed in TSPAN5 knockout cells. (b) Knockdown of TSPAN5 resulted in the downregulation of a series of genes involved in interferon signaling, compatible with the data shown in panels (c) and (d) when cells were exposed to either EtOH or acamprosate.
4/13/2020
1

## Slide 2
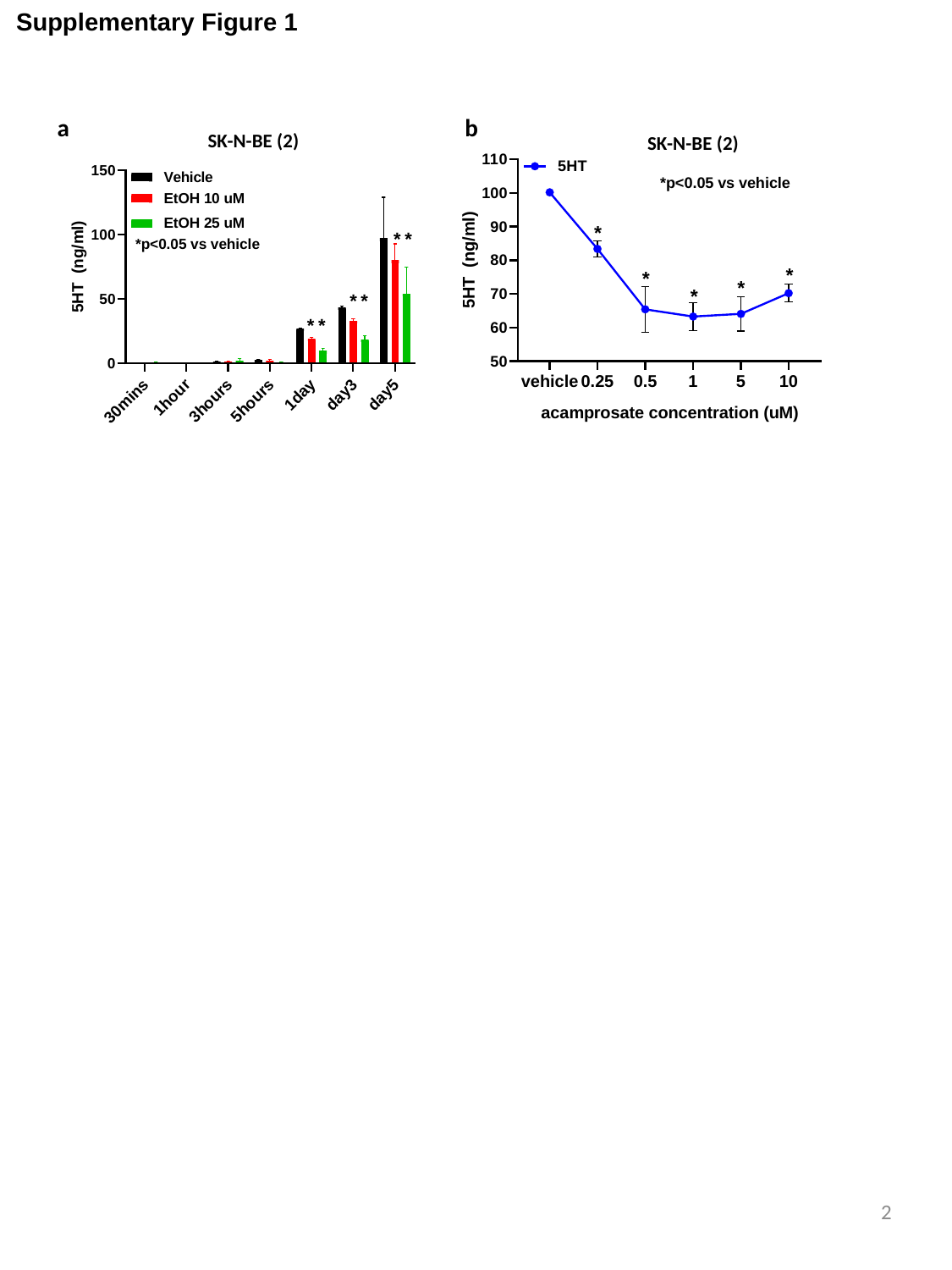

Supplementary Figure 1
a b
SK-N-BE (2)
SK-N-BE (2)
2

## Slide 3
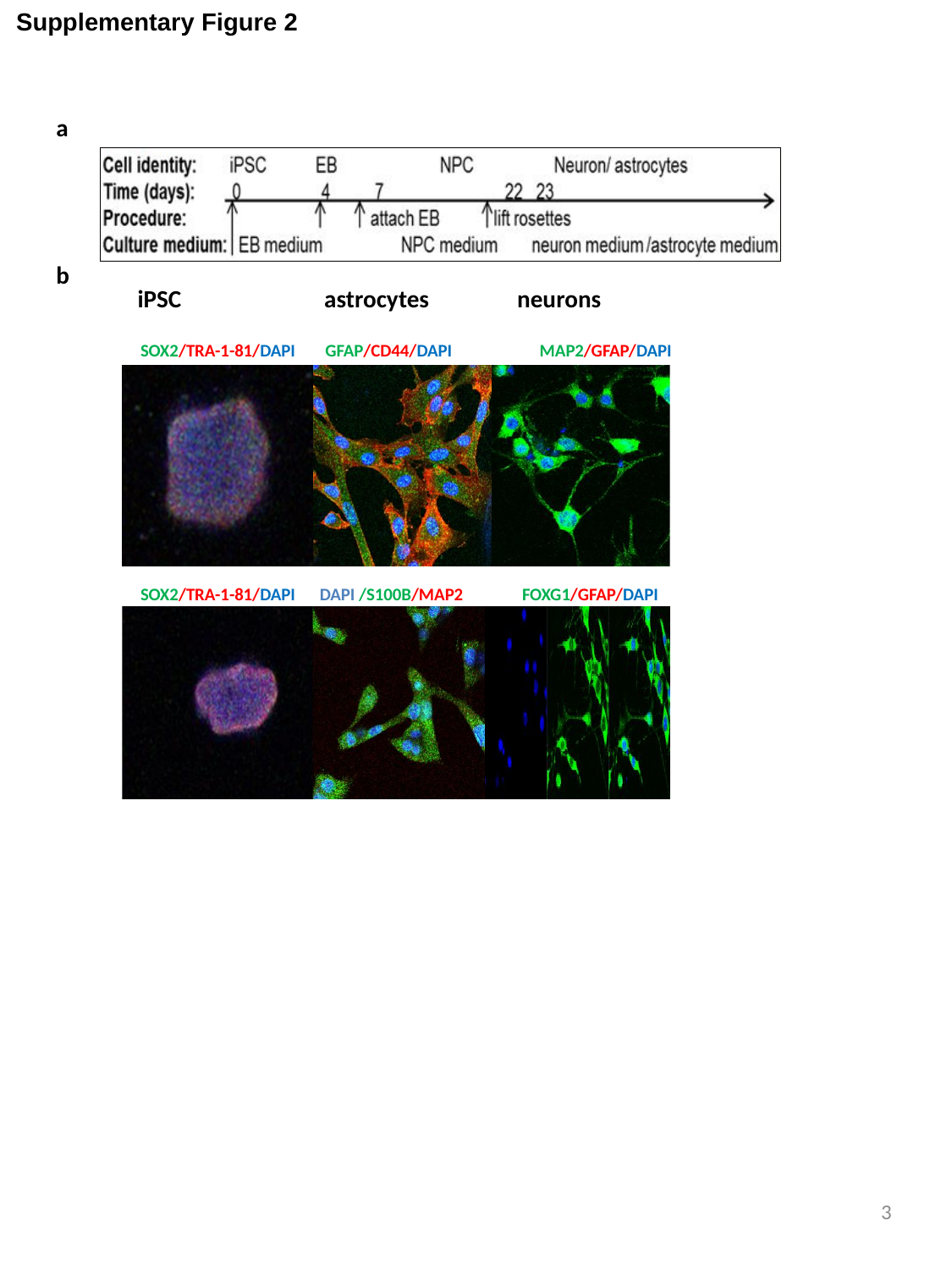

Supplementary Figure 2
a
b
 iPSC astrocytes neurons
SOX2/TRA-1-81/DAPI
GFAP/CD44/DAPI
MAP2/GFAP/DAPI
SOX2/TRA-1-81/DAPI
DAPI /S100B/MAP2
FOXG1/GFAP/DAPI
3

## Slide 4
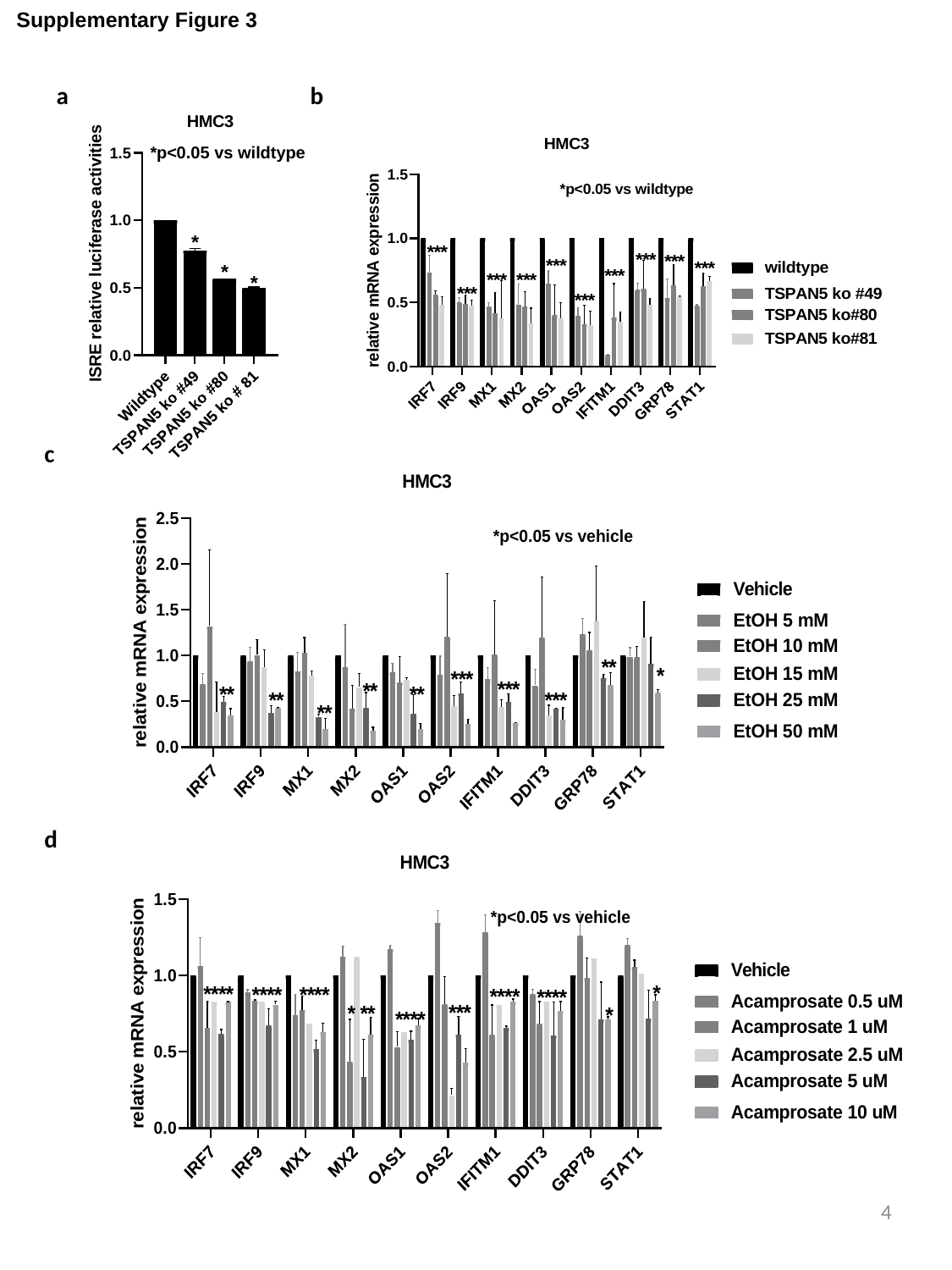

Supplementary Figure 3
a b
c
d
4
